# Purification and properties of glyceraldehyde-3-phosphate dehydrogenase from the skeletal muscle of the hibernating ground squirrel, *Ictidomys tridecemlineatus*

**DOI:** 10.7717/peerj.634

**Published:** 2014-10-28

**Authors:** Ryan A.V. Bell, Jeffrey C. Smith, Kenneth B. Storey

**Affiliations:** Department of Chemistry, Carleton University, Ottawa, Ontario, Canada

**Keywords:** Posttranslational modification, Differential scanning fluorimetry, Torpor, Mass spectrometry, Methylation, Phosphorylation

## Abstract

Glyceraldehyde-3-phosphate dehydrogenase (GAPDH) from the skeletal muscle of euthermic and torpid *Ictidomys tridecemlineatus* was purified to electrophoretic homogeneity using a novel method involving Blue-agarose and Phenyl-agarose chromatography. Kinetic analysis of the enzymes isolated from the two conditions suggested the existence of two structurally distinct proteins, with GAPDH V _max_ being 40–60% less for the enzyme from the torpid condition (in both glycolytic and gluconeogenic directions) as compared to the euthermic enzyme form. Thermal denaturation, in part determined by differential scanning fluorimetry, revealed that purified GAPDH from the torpid animals was significantly more stable that the enzyme from the euthermic condition. Mass spectrometry combined with Western blot analyses of purified GAPDH indicate that the cellular GAPDH population is extensively modified, with posttranslational phosphorylation, acetylation and methylation being detected. Global reduction in GAPDH tyrosine phosphorylation during torpor as well as site specific alterations in methylation sites suggests that that the stable changes observed in kinetic and structural GAPDH properties may be due to posttranslational modification of this enzyme during torpor. Taken together, these results suggest a stable suppression of GAPDH (possibly by some reversible posttranslational modification) during ground squirrel torpor, which likely contributes to the overall reduction in carbohydrate metabolism when these animals switch to lipid fuels during dormancy.

## Introduction

Glyceraldehyde-3-phosphate dehydrogenase (GAPDH; EC 1.2.1.12) is primarily present within the cell as a homotetramer, comprised of approximately 37 kDa subunits. While known to exist as a single isomer, GAPDH has been found to be extensively modified by posttranslational modifications. These include posttranslational phosphorylation (for examples see Tisdale, 2002; Huang et al., 2011), acetylation (Guan & Xiong, 2011; Guarente, 2011; Li et al., 2014), and methylation (Forcina et al., 1971). Many of these modifications are linked to the diverse functions of GAPDH, including roles in membrane trafficking, apoptosis, and autophagy (reviewed in Sirover, 2011; Tristan et al., 2011). These roles are in addition to GAPDH’s canonical role within the glycolytic and gluconeogenic pathways for which it is most well known. In this capacity it converts D-glyceraldehyde-3-phosphate and NAD^+^ to 1,3-bisphosphoglycerate and NADH in the glycolytic pathway, or the reverse reaction in the gluconeogenic pathway. GAPDH’s central role in carbohydrate metabolism makes it an intriguing enzyme to characterize in those animals that experience drastic fluctuations in fuel metabolism. Such is the case with thirteen-lined ground squirrels, which typically shift to non-carbohydrate fuels during winter hibernation (South & House, 1967; Tashima, Adelstein & Lyman, 1970).

Mammalian hibernation is adopted by a select number of mammals in the face of subzero temperatures and dangerously low food availability during prolonged winters. It consists of multiple bouts of aerobic torpor, interspersed with short periods of arousal (denoted interbout arousals). During torpor, the metabolic rate of these animals can be reduced by >95% with a subsequent decrease in body temperature to near ambient (0–5 °C; Geiser, 2004). These bouts of aerobic torpor require many physiological and biochemical changes aimed at suppressing energetically expensive processes and re-establishing a new balance between ATP consumption and production. Of particular importance in this regard are the changes observed in skeletal muscle carbohydrate metabolism during torpor. During dormancy, skeletal muscle glucose catabolism is thought to be suppressed, with lipid oxidation becoming the primary source for energy (South & House, 1967; Tashima, Adelstein & Lyman, 1970). The state of gluconeogenesis on the other hand is somewhat less clear during dormancy. Several studies indicate that there could be an increase or maintenance of skeletal muscle gluconeogenic capacity (Hannon & Vaughn , 1961; el Hachimi et al., 1990; James et al., 2013), while others indicate a decrease in gluconeogenesis during torpor (Zimny & Tyrone , 1957; Galster & Morrison, 1970; Galster & Morrison, 1975). Investigation into the kinetic, physical and regulatory properties of skeletal muscle GAPDH in this study is aimed at clarifying and providing insight into the state of carbohydrate metabolism during ground squirrel torpor.

## Materials and Methods

### Animals

Thirteen-lined ground squirrels (*Ictidomys tridecemlineatus*) were captured in the fall in Illinois and transported to an animal care facility on the National Institutes of Health campus in Bethesda, Maryland. Hibernation experiments were approved by the Animal Care and Use Committee of the National Institute of Neurological Disorders and Stroke (Permit Number ASP 1223-05) and conducted there as previously reported (Mamady & Storey, 2006). Individuals denoted as hibernators were sampled between December and February and only when animals had been in continuous torpor for at least 3 days, as evidenced by continuous low body temperature (∼6 °C) monitored by a subepidermal sensor (denoted as long torpor or LT). Animals that had not entered torpor after at least 3 days in the cold room and showed a continuous high body temperature (36–38 °C) were sampled as euthermic controls (denoted as EC). All animals were sacrificed by decapitation and tissues were quickly excised and frozen in liquid nitrogen. Tissues were then transported by air to Carleton University on dry ice where they were subsequently stored at −80 °C. Ground squirrel hind leg thigh muscle was the tissue taken for skeletal muscle studies.

### Purification of GAPDH

Frozen skeletal muscle samples were homogenized 1:5 w:v in buffer A (25 mM imidazole, pH 7.0, 1.25 mM EDTA, 1.25 mM EGTA, 12.5 mM *β*-glycerophosphate, 5 mM *β*-mercaptoethanol, 5% v:v glycerol, and a few crystals of phenylmethylsulphonyl fluoride added just prior to homogenization) using a Polytron homogenizer. Homogenates were centrifuged at 13,500 g for 30 min at 5 °C, after which supernatants were decanted and held on ice until use in the first purification step.

A blue-agarose column was prepared with the dimensions of 1 × 3.5 cm (diameter × height) and equilibrated in 15 mL of buffer A. A 1.5 mL aliquot of crude muscle extract was then loaded on the column, which was then washed with 30 mL of buffer A. This was sufficient to reduce the absorbance of the fractions at 280 nm to that of the buffer alone. GAPDH was then eluted with a 0–1 M KCl gradient in buffer A and activity was assayed under *V*_max_ conditions. The top three activity fractions were pooled and applied to a phenyl-agarose column.

The phenyl-agarose column was prepared with the same dimensions as for the blue-agarose column, and equilibrated in 15 mL buffer B (buffer A containing 1 M KCl). Salt was also added to the pooled enzyme from the blue-agarose column to bring the concentration to approximately 1 M KCl, and then the enzyme was applied to the phenyl-agarose column. The column was washed with 30 mL of buffer B, which was sufficient to wash away any unbound protein. GAPDH was eluted with a linear 1–0 M KCl gradient (i.e., declining salt concentration) and assayed under *V*_max_ conditions. The three top activity fractions were pooled and held at 4 °C until use.

### GAPDH assay

GAPDH was assayed spectrophotometrically at 340 nm using a Thermo Labsystems Multiskan spectrophotometer. Optimal assay conditions for the oxidation of D-glyceraldehyde-3-phosphate (GAP) were 2.5 mM GAP, 1.5 mM NAD^+^, 15 mM Na_2_HAsO_4_, and 50 mM NaH_2_PO_4_, pH 8.0. Optimal assay conditions for the reverse reaction were 2.5 mM 3-phosphoglycerate (3PG), 2 mM Mg-ATP, 0.1 mM NADH, 1 unit of phosphoglycerate kinase, and 50 mM Tris–HCl buffer, pH 8.0. Regardless of the assay direction, the enzyme reaction was initiated by the addition of 10 µL of purified GAPDH protein.

It is important to note that in assessing GAPDH activity in the 1,3-bisphosphoglycerate (1,3BPG)-reducing direction that commercial 3-phosphoglycerate kinase was used to rapidly convert 3-phosphoglycerate into 1,3BPG for use in the GAPDH reaction. Thus, kinetic parameters for 1,3BPG were measured indirectly in this study (although phosphoglycerate kinase activity was always in high excess in the assay).

Microplate Analysis (MPA) and Kinetics computer programs were used to analyze the data and determine Michaelis constants (*K_m_*) for substrates and inhibitor concentrations that reduced enzyme activity by 50% (*I*_50_) values (Brooks, 1992; Brooks, 1994). GAPDH activity is presented as units/mg soluble protein with 1 Unit is defined as the amount of enzyme necessary to produce 1 µmol of NADH or NAD^+^ per minute at room temperature. Soluble protein was measured using the Coomassie Brilliant Blue dye binding method with the Bio-Rad prepared reagent and a standard curve created using bovine serum albumin.

### Effect of temperature on GAPDH activity

To assess the effect of temperature on GAPDH activity, various kinetic parameters were determined at low (5 ± 1 °C) and high (36 ± 1 °C) temperature. To establish the low temperature condition, the Thermo Labsystems Multiskan spectrophotometer and the microplates containing all solutions except for purified GAPDH (being cooled separately) were placed inside a temperature-controlled incubator set to 5 °C. To establish the high temperature conditions, the microplate chamber of the Thermo Labsystems Multiskan spectrophotometer was set to warm to 36 °C and the microplates (containing all solutions except for the purified GAPDH) were warmed on a Echo Thermomicroplate heater (Torrey Pines Scientific). It is important to note that for assays at 36 °C the enzyme was not allowed to equilibrate at this high temperature due to the lack of stability of GAPDH when heated (experimentally identified in this study). Prior to assay, the well temperature was monitored by a digital telethermometer placed inside the well. The final temperature of the well was also determined after the assay to ensure a stable reaction temperature.

The thermal stability of GAPDH was assessed in a manner similar to that described by Iddar et al. (2003). Briefly, aliquots of purified GAPDH were incubated in a water bath at a given temperature for 15 min, after which they were immediately chilled on ice and assayed under optimal conditions at 25 °C.

The effects of temperature on GAPDH were also analyzed using Arrhenius plots to determine GAPDH activation energy (*E_a_*). The maximal velocities (*V*_max_) of GAPDH in the 1,3BPG-reducing reaction were measured at 5 °C intervals from 5–35 °C. Assays at temperatures below or above 25 °C (room temperature) were conducted as described above for the 5 °C and 36 °C assays, respectively. Well temperature was again confirmed using a telethermometer at the end of each assay.

### Differential scanning fluorimetry

Differential scanning fluorimetry (DSF) is a high throughput method that monitors the thermal unfolding of proteins in the presence of a fluorescent dye (Niesen, Berglund & Vedadi, 2007). Prior to DSF, purified EC and LT GAPDH were spun through a G50 Sephadex column, pre-equilibrated in DSF buffer (100 mM potassium phosphate, pH 7.0, 150 mM NaCl), at 2500 rpm for 1 min. Protein samples were then aliquoted to a concentration of 0.05 µg/µL/well into the wells of a 48-well, thin-walled PCR plate along with the dye SYPRO Orange (5X final concentration of the dye). PCR plates were then sealed with sealing tape and placed into a BioRad iCycler5 PCR instrument. SYPRO Orange (Invitrogen) fluorescence was monitored as described by Biggar and colleagues (2012). Briefly, SYPRO Orange was excited through the transmission of light through the FAM filter (485 ± 30 nm), with the subsequent emission of light through the ROX filter (625 ± 30 nm). Measurements were taken every 30 s in 1 °C increments from 25 °C to 97 °C. Subsequent analysis of the fluorescent data using OriginPro 8.5 and the Boltzmann distribution curve yielded the midpoint temperature of the protein-unfolding transition, known as the protein melting temperature (*T_m_*), for GAPDH.

### GAPDH digestion and mass spectrometry

Purified GAPDH samples were digested in 50 mM ammonium bicarbonate containing sequencing grade porcine trypsin at 0.013 µg/µL (Promega). Extracted peptides were dried down in a vacuum apparatus overnight followed by resuspension in 30 µL of 0.1% formic acid for use in subsequent mass spectrometry. Mass analysis was carried out on an ESI-TOF QUAD-MS/MS mass spectrometer. Samples were loaded onto a 5 µm × 50 mm column packed in-house with 5 µm C18 beads (100 Å pore size) (Michrom Bioresources, CA) at a rate of 300 µL/min using a Dinoex 3000 ultimate HPLC system (Thermo Fisher Scientific, IL). Peptides were eluted using a 0–60% (v/v) gradient of acetonitrile with 0.1% formic acid over 30 min. Mass spectra were analyzed using Mascot (Matrix Science). Mascot MS/MS ion search was conducted with the following parameters: allowance of two missed cleavages by trypsin, fixed modification of carbamidomethyl, peptide tolerance of 100 ppm, fragment mass tolerance of 100 ppm, and a peptide charge of 2+, 3+, or 4+. Peptides were only selected if the associated fragmentation patterns displayed three consecutive y or b ions.

### SDS polyacrylamide gel electrophoresis, immunoblotting, and ProQ diamond phosphoprotein staining

Purified EC and LT GAPDH samples were mixed 2:1 (v:v) with SDS loading buffer (100 mM Tris buffer, pH 6.8, 4% w:v SDS, 20% v:v glycerol, 0.2% w:v bromophenol blue, and 10% v:v 2-mercaptoethanol) and boiled for 5 min, cooled on ice and frozen at −20 °C until use.

SDS resolving gels (15% v/v acrylamide, 400 mM Tris, pH 8.8, 0.1% w/v SDS, 0.2% w/v ammonium persulfate [APS], 0.04% v/v TEMED) were prepared with a 5% stacking gel (5% acrylamide, 190 mM Tris, pH 6.8, 0.1% w/v SDS, 0.15% w/v APS, 0.1% v/v TEMED). Purified EC and LT GAPDH were loaded onto these gels and separated electrophoretically in SDS-PAGE running buffer (25 mM Tris-base, 190 mM glycine, and 0.1% w/v SDS) at 180 V for 45 min. A 3 µL aliquot of Spectra™ Multicolor Broad Range Protein Ladder was added to one lane of every gel to provide molecular weight markers. Commercial pure rabbit muscle GAPDH (Boehringer Mannheim) was also loaded onto the gel to confirm the correct location of GAPDH subunits. Following electrophoresis, proteins were either electroblotted onto polyvinylidiene difluoride (PVDF) membranes (Millipore) by wet transfer and used for immunoblotting or fixed and stained with ProQ diamond phosphoprotein stain.

With regards to phosphoprotein staining, the SDS-gel containing purified EC and LT GAPDH was washed three times in fixing solution (50% v:v methanol, 10% v:v acetic acid) and subsequently left overnight at 4 °C in this solution. The following day the gel was washed three times for ten minutes each time in ddH_2_O, after which the gel was immersed in ProQ Diamond phosphoprotein stain (Invitrogen, Eugene, OR) for 30 min. Once the stain was placed onto the gel, the gel remained covered in aluminum foil to protect the light sensitive stain. After staining, the gel was washed three times for 10 min each time with ddH_2_O before being viewed in a ChemiGenius Bioimaging System (Syngene, Fredrick, MD). The fluorescent bands were then quantified using GeneTools software. Subsequently, the SDS-gel was stained with Coomassie blue (0.25% w:v Coomassie Brilliant Blue R in 50% v/v methanol, 7.5% v:v acetic acid). The GAPDH ProQ band intensities were subsequently standardized against the Coomassie blue-stained GAPDH band intensities to negate errors caused by sample loading.

Immunoblotting began with the wet transfer of proteins from 15% SDS gels (run as above) onto PVDF membranes previously soaked in methanol. Electroblotting was performed at room temperature for 1.5 h at 160 mA under transfer buffer (25 mM Tris, pH 8.5, 192 mM glycine, and 20% v/v methanol). Following protein transfer, PVDF membranes were incubated overnight at 4 °C with one of the following primary antibodies that detect specific posttranslational modifications to the enzyme: (1) Pan-acetyl (Santa Cruz Biotechnology), (2) Phospho-serine (Calbiochem), (3) Phospho-threonine (Invitrogen), (4) Phospho-tyrosine (Invitrogen), or (5) Methyl-lysine (StressMarq Bosciences Inc.). All primary antibodies were diluted 1:1000 v:v in Tris-buffered saline with Tween-20 (TBST; 20 mM Tris-base, 140 mM NaCl, 0.05% Tween-20) with a small amount of sodium azide added. After the overnight incubation, membranes were washed with TBST three times for 5 min each, followed by incubation with the appropriate secondary antibody conjugated with horseradish peroxidase (Bioshop Canada) at a dilution of 1:4000 v:v in TBST. Membranes were incubated at room temperature for 1.5 h, and then washed three times for 5 min each time with ddH_2_O. Signal was then detected using enhanced chemiluminescence (ECL), initiated by the addition of 600 µL of hydrogen peroxide and 600 µL of luminol reagent to the membrane’s surface for several seconds. The mixture was then poured off and the chemiluminescence was detected with the ChemiGenius Bioimaging System (Syngene, MD, USA). Membranes were then stained with Coomassie blue (0.25% w/v Coomassie Brilliant Blue R in 50% methanol, 7.5% acetic acid) and destained with destaining solution (60% v/v methanol, 20% v/v acetic acid in ddH_2_O) until bands were clearly seen. Band densities were analyzed using the associated GeneTools software, and were normalized against with the corresponding Coomassie blue stained bands.

### In vitro incubations that stimulate protein kinases or protein phosphatases

Crude muscle extracts were prepared as described above, and then subsequently filtered through a small G50 Sephadex column that had been pre-equilibrated in buffer B (25 mM imidazole, 5% v:v glycerol, 5 mM 2-mercaptoethanol, pH 7.0). Aliquots of the filtered supernatants were incubated overnight at 4 °C with specific inhibitors and stimulators of protein kinases or protein phosphatases, as described in MacDonald & Storey (1999). Each aliquot was mixed 1:2 v:v with buffer B plus the following additions:

(1) Stop conditions: 1.25 mM EGTA, 1.25 mM EDTA, and 12.5 mM ß-glycerophosphate.
(2) Protein Tyrosine Phosphatases (PTP)—30 mM NaF
(3) Alkaline Phosphatase (Alk PPase)—10 mM MgCl_2_ + 5 mM EDTA + 1 U calf intestine alkaline phosphatase per incubation tube.
(4) Protein Kinase C (PKC)—1.3 mM CaCl_2_ + 7 µg/mL phorbol 12-myristate 13-acetate (PMA) + 5 mM ATP + 5 mM MgCl_2_ + 30 mM NaF + 5 mM Na_3_V O_4_
(5) AMP-activated Protein Kinase (AMPK)—1 mM AMP + 5 mM ATP + 10 mM MgCl_2_ + 30 mM NaF + 5 mM Na_3_V O_4_.
(6) Calcium/calmodulin Activated Protein Kinase (CaMK)—1 U of calmodulin/incubation tube + 1.3 mM CaCl_2_ + 5 mM ATP + 10 mM MgCl_2_ + 30 mM NaF + 5 mM Na_3_V O_4_.

Samples were spun at 2500 RPM for 1 min through a G50 Sephadex column that had been pre-equilibrated in buffer A. This removed small molecular weight molecules and allowed GAPDH activity to be reassessed in the GAP-oxidizing direction under optimal conditions.

### GAPDH bioinformatics

The nucleotide and amino acid sequence of 13-lined ground squirrel GAPDH was deduced using multiple GAPDH sequences from a variety of animals. As the basis for the construction, the rat (*Rattus norgevicus*) GAPDH coding nucleotide sequence (NCBI Reference Sequence: NM_017008.3) was used in the Basic Local Alignment Search Tool (BLAST) to mine whole genome shotgun sequences from the 13-lined ground squirrel genome, and the sequence with the highest coverage was taken (Accession Number: AAQQ01785922.1). This sequence was then compared to three other whole genome shotgun sequences of 13-lined ground squirrel GAPDH as well as a low-coverage 1.9X 13-lined ground squirrel GAPDH sequence produced by the Broad Institute (http://www.ensembl.org/Spermophilus_tridecemlineatus/Info/Index). When discrepancies existed between the whole genome shotgun reads and the Ensemble sequence, typically, the whole genome shotgun sequence was taken as true. However, if there were discrepancies between whole genome shotgun reads themselves as well as the ensemble sequence, then GAPDH sequences from the rat, mouse, pig and cow were used to help decipher the most likely nucleotide sequence. Sequences were compared using DNAMAN Version 4.0 (Lynnon BioSoft).

The above amino acid sequence and Scansite 2.0 (Obenauer, Cantley & Yaffe, 2003) were used to assess potential phosphorylation sites and specific modifiers of GAPDH phosphorylation state. The predictions were made under medium stringency.

## Results

### Purification of GAPDH

**Figure 1:**
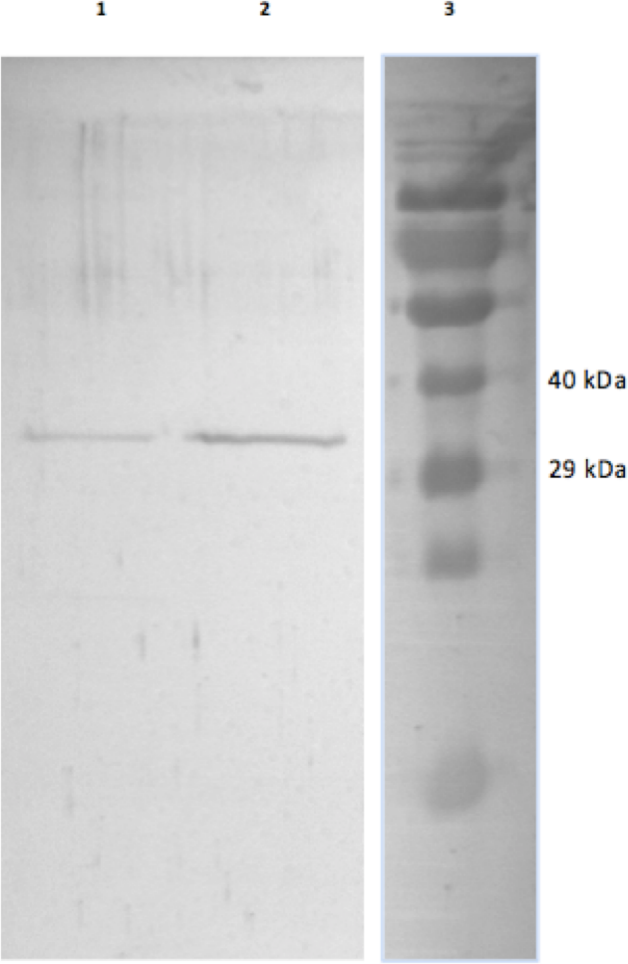
Purified EC and LT skeletal muscle GAPDH. The SDS-gel was stained with Coomassie Brilliant blue and the lanes show (1) purified LT GAPDH (2) purified EC GAPDH and (3) Spectra Multicolour protein ladder with the 40 kDa and 29 kDa band indicated on the right.

GAPDH from skeletal muscle of 13-lined ground was purified using a Blue-agarose affinity column followed by a hydrophobic column, phenyl-agarose. The overall yield of the final EC GAPDH preparation was 45% with a fold purification of 460 and a final specific activity of 146 U/mg (Table 1A). The overall yield of LT GAPDH was 22% with a fold purification of 230 and a specific activity of 100 U/mg. This purification procedure yielded electrophoretically pure preparations that were used in all subsequent experiments (Fig. 1).

**Table 1:** Purification scheme for 13-lined ground squirrel (A) EC and (B) LT GAPDH.

Purification step	Total protein (mg)	Total activity (U)	Specific activity (U/mg)	Fold purification	% Yield
**A**					
Supernatant	14.4	4.9	0.34	–	–
Blue Agarose	1.4	5.0	3.6	10	100
Phenyl Agarose	0.015	2.2	146	430	45
**B**					
Supernatant	8.1	3.6	0.44	–	–
Blue Agarose	1.7	2.5	1.5	3.4	72
Phenyl Agarose	0.008	0.8	100	230	22

### GAPDH kinetics

The activity of GAPDH in both directions was investigated at two temperatures, one that corresponded to approximately euthermia (36 °C) and one typical of torpor (5 °C). Several significant differences in GAPDH kinetics were observed between EC and LT GAPDH under high and low temperatures. At 5 °C, the *V*_max_ values in the GAP-oxidizing direction and 1,3BPG-reducing directions were ∼63% and 44% less, respectively, for LT GAPDH as compared to EC GAPDH (Table 2). At the same temperature, the *K_m_* NAD^+^ for LT GAPDH was 60% lower than that of EC GAPDH. At 36 °C, GAPDH *V*_max_ in the GAP-oxidizing direction was significantly different between EC and LT purified samples, LT GAPDH *V*_max_ being ∼67% less as compared to EC GAPDH. Similar to the situation at 5 °C, the *K_m_* NAD^+^ was 60% lower for LT GAPDH as compared to the euthermic value (Table 2). *K_m_* GAP and *K_m_* 1,3BPG did not differ between GAPDH from EC and LT conditions at either temperature.

**Table 2:** Kinetic analysis of EC and LT GAPDH in the GAP-oxidizing and 1,3BPG-reducing directions at 5 °C and 36 °C. Data are means ± SEM, *n* ≥ 3 independent determination on separate enzyme preparations. *K_m_* values were determined at optimal co-substrate concentrations (as indicated in Materials and Methods).

	5 °C		36 °C	
	EC	LT	EC	LT
*K_m_* GAP (mM)	0.45 ± 0.02	0.52 ± 0.07	1.4 ± 0.2	1.5± 0.2
*K_m_* NAD^+^ (mM)	1.3 ± 0.2	0.47 ± 0.06^*^	1.3 ± 0.3	0.5 ± 0.1^*^
*K_m_* 1,3BPG (mM)	0.22 ± 0.03	0.28 ± 0.03	0.24 ± 0.02	0.24 ± 0.03
*V*_max_ (U/mg; GAP oxidation)	30 ± 5	11.4 ± 0.9^*^	150 ± 18	50 ± 2^*^
*V*_max_ (U/mg; 1,3BPG reduction)	32 ± 4	18 ± 2^*^	155 ± 5	130 ± 13

**Notes.**

* indicates that the LT value is significantly different from the control (EC) value at the same temperature as determined using the Student’s *t*-test, *p* < 0.05.

The maximal activity of GAPDH was also monitored over a range of temperatures between 5 °C and 35 °C to determine the activation energy (*E_a_*) for the 1,3BPG-reducing reaction of GAPDH. The calculated *E_a_* for LT GAPDH was 2-fold higher than the corresponding value for EC GAPDH (Table 3).

**Table 3:** Physical properties of skeletal muscle GAPDH from euthermic and torpid 13-lined ground squirrels. Data are means ± SEM, *n* ≥ 4 independent determinations on separate enzyme preparations. Activation energy was measured for the 1,3BPG-reducing reaction of GAPDH. I_50_ values were determined for the GAP-oxidizing reaction at optimal substrate concentrations (as indicated in Materials and Methods).

	EC	LT
Activation energy (KJ/mol)	28 ± 2	54 ± 2^*^
*T_m_* (°C)	54.7 ± 0.1	56.1 ± 0.2^*^

**Notes.**

* indicates that the LT value is significantly different from the control (EC) value as determined using the Student’s *t*-test, *p* < 0.05.

### GAPDH stability and effectors

**Figure 2:**
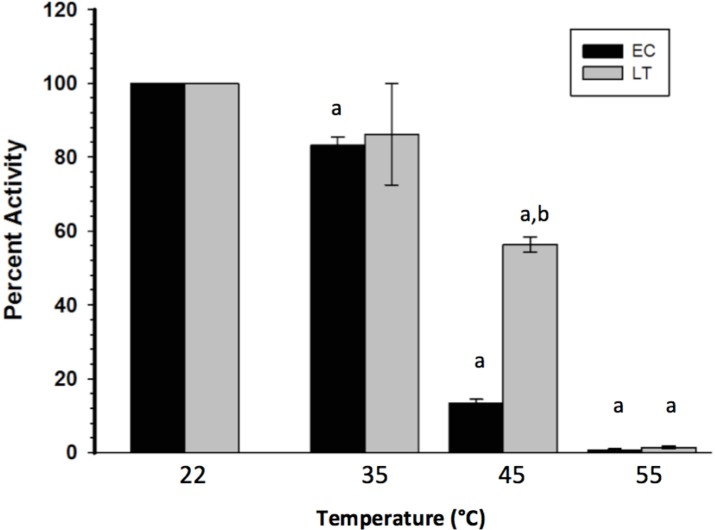
Heat-induced inactivation of EC and LT skeletal muscle GAPDH. Data are means ± SEM, *n* = 4 independent determinations on separate enzyme preparations. a—The value is significantly different from the value for the same condition assayed at 22 °C, as determined by a Holm–Sidak test, *p* < 0.05. b—The LT value is significantly different from the EC value at the same temperature, as determined by the Holm–Sidak test, *p* < 0.05.

To assess the thermal stability of each enzyme form, the purified extracts were subjected to short (15 min) incubations at different temperatures, after which GAPDH was assayed at room temperature. Figure 2 indicates that LT GAPDH appears to be significantly less susceptible to thermal denaturation as compared to EC GAPDH, with 45 or 55 °C temperatures being required to cause a significant decrease in LT GAPDH enzyme activity as compared to 35 and 45 °C for EC GAPDH. Furthermore, LT GAPDH displayed a significantly higher thermal melting point of 56.1 ± 0.2 °C as compared to the euthermic GAPDH *T_m_* of 54.7 ± 0.1 °C (Table 3).

### GAPDH posttranslational modification

**Figure 3:**
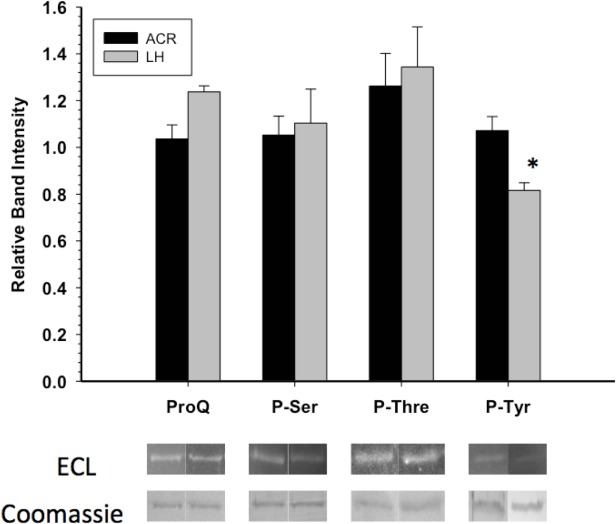
Phosphorylation state of skeletal muscle EC and LT GAPDH. Total phosphorylation was determined by ProQ Diamond phosphoprotein staining, and the relative levels of P-Ser, P-Thr and P-Tyr were determined with phospho-specific antibodies. Data are means ± SEM, *n* ≥ 3 determinations on independent enzyme samples. * indicates a significant difference in GAPDH phosphorylation between EC and LT conditions using the Student’s *t*-test, *p* < 0.05.

GAPDH posttranslational modification was primarily assessed through Western blots and phosphoprotein staining. Western blotting with antibodies detecting phospho-serine or phospho-threonine indicated no significant difference in these PTMs between EC and LT GAPDH (Fig. 3). This coincided with the assessment of the overall phosphorylation state of GAPDH by ProQ Diamond phosphoprotein staining which indicated no significant difference in total protein-bound phosphate between EC and LT conditions. However, GAPDH from the two conditions differed significantly in phospho-tyrosine content, with LT GAPDH showing 35% less phosphorylation as compared to the euthermic enzyme (Fig. 3).

**Figure 4:**
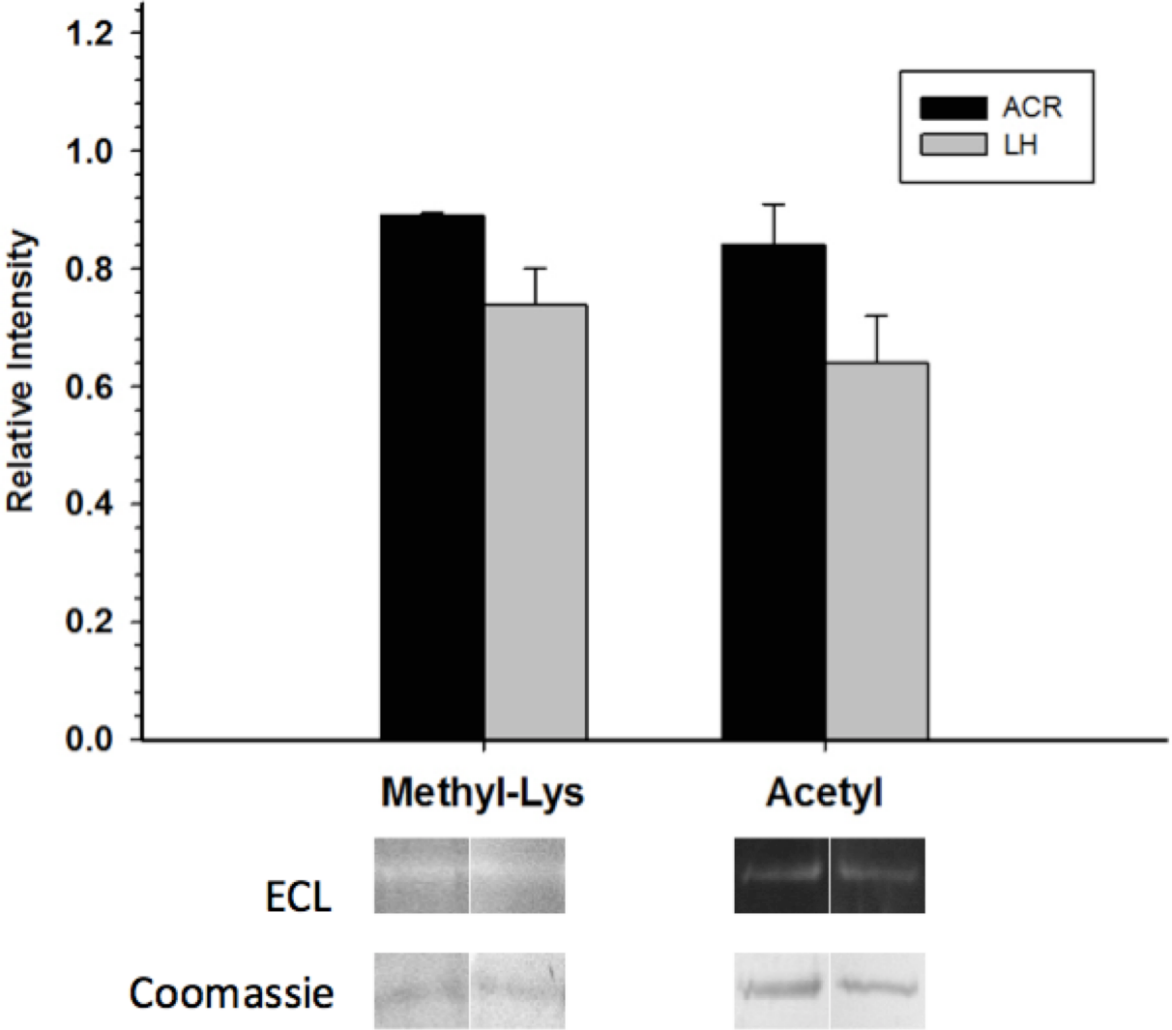
Posttranslational methylation and acetylation of EC and LT skeletal muscle GAPDH. The relative changes in protein methylation and acetylation, as determined by immunoblotting. Data are means ± SEM, *n* = 3 independent samples run on the same gel. * Indicates a significant difference between EC and LT GAPDH as determined using the Student’s *t*-test, *p* < 0.05.

Western blots looking at the overall acetylation level of purified GAPDH showed no significant difference between control and torpid enzyme forms (Fig. 4). The same results were seen when probing purified GAPDH with anti-methyl-lysine antibody (i.e., no significant differences between EC and LT conditions). Interestingly, analysis of tryptic peptides of purified GAPDH by ESI-QUAD TOF MS/MS revealed several peptides that have been lysine-methylated. A GAPDH peptide from the LT condition displayed a dimethylated lysine residue (K213). The same peptide from the EC condition also displayed this same dimethylation modification, but was also detected in a monomethylation form (Table 4). In addition to lysine-methylation a single peptide from GAPDH from the EC condition contained a methylated aspartate residue (D242). This peptide was not detected in the GAPDH from the LT condition. It should be noted that the sequence coverage for LT GAPDH was substantially lower than that for the control enzyme; approximately 11% for LT GAPDH as compared to 35% for EC GAPDH (Appendix S1A and S1B).

**Table 4:** The peptides detected by mass spectrometry in ground squirrel muscle GAPDH from EC versus LT conditions. The numbers preceding and following each peptide indicate the residue number in the linear GAPDH amino acid sequence (Appendix S1A and S1B). Underlined residues are those that have been detected to be posttranslationally modified (modification is given at the end of the peptide sequence). Details of mass spectrometry peptide selection are given in the Materials and Methods.

EC	LT
2-VKVGVDGFGR-11	2-VKVGVDGFGR-11
60-AENGKLVINGK-70	60-AENGKLVINGK-70
71-SISIFQER-78	
71-SISIFQERDPANIK-84	
144-IVSNASCTTNCLAPLAK-160	
199-GAAQNIIPASTGAAK-213	199-GAAQNIIPASTGAAK-213
199-GAAQNIIPASTGAAK-213 + Methyl (K)	
199-GAAQNIIPASTGAAK-213 + Dimethyl (K)	199-GAAQNIIPASTGAAK-213 + Dimethyl (K)
218-VIPELNGK-225	
233-VPTPNVSVVDLTCR-246	
233-VPTPNVSVVDLTCRLEK-246 + Methyl (DE)	
233-VPTPNVSVVDLTCRLEK-246	
262-QASEGPLK-269	
308-LISWYDNEFGYSNR-321	

### GAPDH regulation by reversible phosphorylation

**Figure 5:**
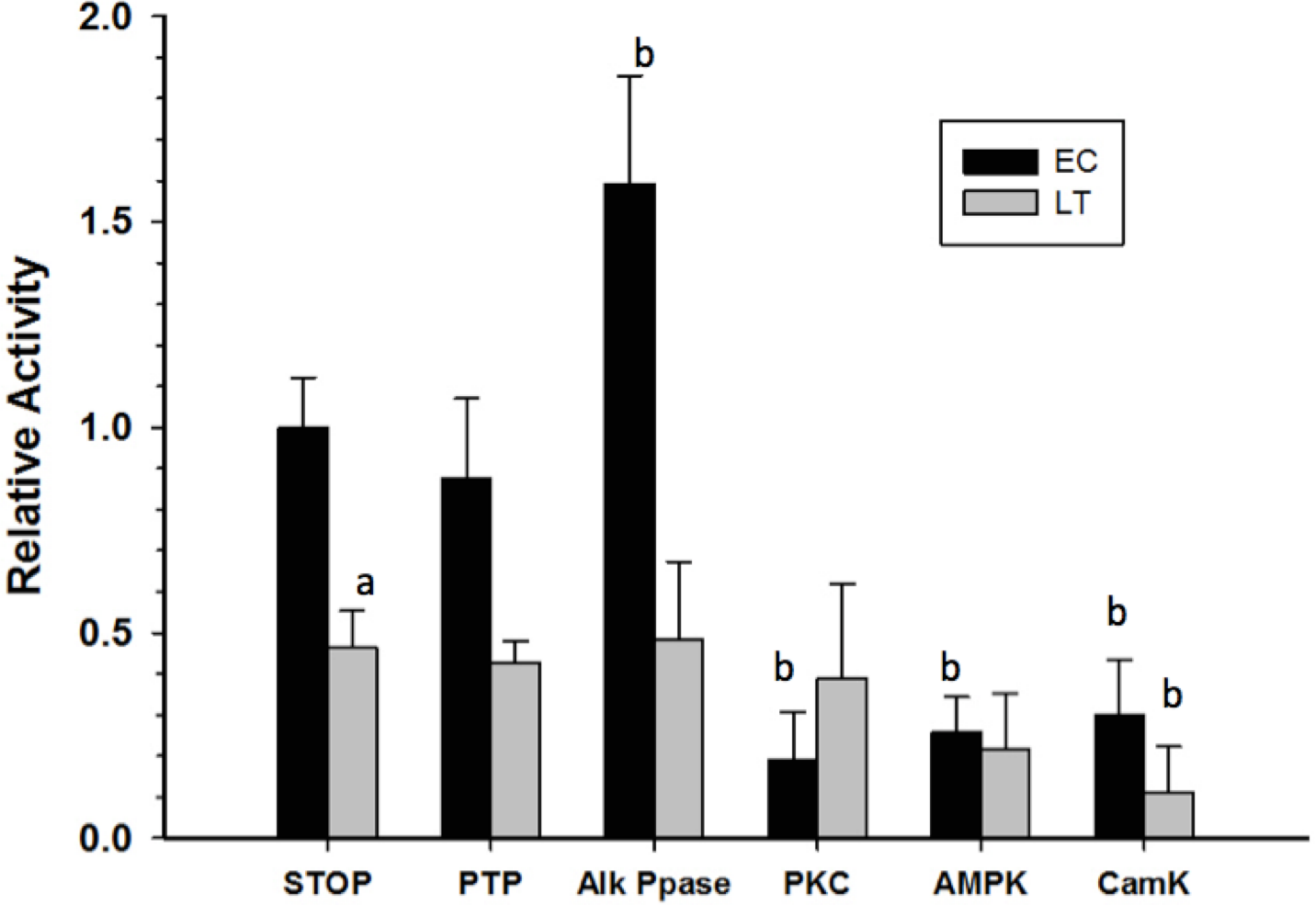
Effect on GAPDH activity of *in vitro* incubations that stimulate protein kinases or protein phosphatases. Data are means ± SEM, *n* = 3 determinations on independent enzyme samples. a—Indicates a significant difference between EC and LT GAPDH activities under the STOP condition, as determined by the Student’s *t*-test, *p* < 0.05. b—Indicates a significant difference between the GAPDH activity value and the corresponding STOP condition, as determined by the Dunnett’s test, *p* < 0.05.

Crude skeletal muscle samples were incubated in solutions that either stimulated the activities of selected endogenous protein kinases/phosphatases or in a solution that activated commercial alkaline phosphatase, and then GAPDH activity was reassessed at room temperature. Incubations that aimed to stimulate endogenous tyrosine phosphatases (PTP) did not have any significant effect on GAPDH activity while the action of commercial alkaline phosphatase (Alk Ppase) caused a 50% increase in EC GAPDH activity when compared to EC GAPDH held in STOP buffer (endogenous kinases and phosphatases both inhibited) (Fig. 5). Conversely, incubations that stimulated activities of protein kinase C (PKC), AMP-dependent protein kinase (AMPK), or Calcium-calmodulin protein kinase (CaMK) lead to ∼80% decreases in EC GAPDH activity as compared to the activity under STOP condition. Only incubation under conditions that stimulated CaMK altered the activity of LT GAPDH, the relative activity decreasing by ∼60% compared with STOP conditions.

## Discussion

GAPDH is a multifunctional enzyme that plays a role in many cellular processes, with its most critical role being its central position in the glycolytic and gluconeogenic pathways within the cell. Its role in carbohydrate metabolism makes it an intriguing enzyme to investigate in animals that experience alterations in fuel metabolism due to drastic environmental changes. Thirteen-lined ground squirrels endure low-temperature torpor during their winter hibernation months and experience drastic changes to cellular metabolism, which may include reductions in both glycolytic and gluconeogenic rates (Galster & Morrison, 1970; Galster & Morrison, 1975; Abnous & Storey, 2008). The present study indicates that regulation of GAPDH activity in both glycolytic and gluconeogenic directions may contribute to the suppression of carbohydrate metabolism during torpor.

Ground squirrel muscle GAPDH was purified to electrophoretic homogeneity by using a combination of affinity and hydrophobic chromatography. The end result was a yield of purified enzyme of 22% for LT GAPDH and 45% for EC GAPDH (Tables IA and IB). This purification scheme appears on-par to that used in several other recent purifications of muscle GAPDH (see Fourrat, Iddar & Soukri, 2007; Baibai et al., 2007). Thus, the purification procedure presented here indicates a novel and effective method of isolating GAPDH from a complex protein mixture.

Kinetic analysis of GAPDH purified from EC versus LT animals revealed several differences between the two enzyme forms. Properties of the enzymes were compared at two temperatures, a high temperature (36 °C) that is very close to euthermic *T_b_* and a low temperature (5 °C), that is approximately the minimum *T_b_* that squirrels experience during torpor (i.e., if ambient temperature in the burrow falls to subzero values, squirrels respond with low rates of thermogenesis to keep *T_b_* above 0 °C). A comparison of EC and LT GAPDH revealed a number of interesting features. At both high and low temperatures, the *K_m_* NAD^+^ was substantially lower for LT GAPDH as compared to EC GAPDH (Table 2). Furthermore, GAPDH *V*_max_, in both GAP-oxidizing and 1,3-BPG-reducing directions was significantly reduced for the enzyme isolated from torpid animals (Table 2). Since *in vivo* concentrations of NAD^+^ likely meet or exceed the levels necessary for maximal GAPDH velocity (White & Schenik, 2012), it may be that the differences in *V*_max_ are most important in assessing the functioning of GAPDH during euthermia and torpor. The decrease in LT GAPDH functionality is also corroborated by the near doubling of its activation energy as compared to the control enzyme (Table 3). In this respect the results for GAPDH at 5 °C do coincide with the expected reduction in skeletal muscle glycolysis as the ground squirrel’s transition to utilizing lipids as their primary energy source (South & House, 1967; Tashima, Adelstein & Lyman, 1970). The results presented here also support the decrease in gluconeogenesis that was suggested by decreased glycogen levels measured previously (Zimny & Tyrone , 1957; Galster & Morrison, 1970; Galster & Morrison, 1975). These kinetic analyses are also supported by a previous study of skeletal muscle GAPDH from the hibernating Jerboa (*Jaculus orientalis*; Iddar et al., 2003), which indicated a similar decrease in GAPDH activity during torpor. Thus, the reduced GAPDH function may play a role in slowing muscle glycolytic and gluconeogenic rates during torpor.

Kinetic changes in an enzyme originating from different physiological states are often indicative of structural differences between the enzymes. These variations can be confirmed through structural studies that probe the enzyme’s susceptibility to denaturation/inactivation using temperature as a perturbant. Thermal denaturation of EC and LT GAPDH indicated a distinct difference between the two enzyme forms with LT GAPDH being able to withstand incubation at higher temperatures as compared to EC GAPDH (Fig. 2). The greater LT GAPDH thermostability was corroborated by differential scanning fluorimetry, which demonstrated that LT GAPDH had a *T_m_* that was several degrees above that for EC GAPDH (Table 3). This resistance to thermal denaturation by GAPDH from the hibernating ground squirrel was similar to that seen by Iddar et al. (2003), who investigated purified skeletal muscle GAPDH from the hibernating Jerboa (*Jaculus orientalis*). These studies give a clear indication that the enzyme from euthermic and torpid conditions are structurally distinct.

Given that GAPDH is well known to be present as a single isozyme, the above structural differences between EC and LT GAPDH are likely due to some posttranslational modification of the protein. Analysis of GAPDH phosphorylation state through ProQ Diamond phosphoprotein staining and phospho-specific antibodies indicated that differential tyrosine phosphorylation may be the mechanism by which GAPDH activity is altered during torpor; decreased GAPDH tyrosine phosphorylation being correlated with decreased enzyme activity (Table 2 and Fig. 3). In an attempt to assess whether tyrosine phosphorylation affects GAPDH activity, crude homogenates were incubated in conditions that would allow tyrosine phosphatases to function, after which GAPDH activity was reassessed. Figure 5 indicates that no significant changes in GAPDH activity were observed when tyrosine phosphatases were allowed to act, suggesting that tyrosine phosphorylation may not be responsible for the activity changes measured in this study. However, stimulation of several protein serine/threonine kinases (PKC, AMPK and/or CaMK) or alkaline phosphatase did generate significant changes in GAPDH activity; enzyme phosphorylation causing a significant decrease in GAPDH activity (Fig. 5). This suggests that GAPDH phosphorylation state does play a significant role in regulating its activity, however key site-specific differences in EC and LT GAPDH phosphorylation state may be too small to be determined by the broad methods used in this study. To this end, mass spectrometry was conducted in an attempt to identify phospho-peptides that may be differentially modified between the two conditions. No phospho-peptides were conclusively identified, and thus a more extensive investigation into the site-specific phosphorylation events would need to be done to conclusively determine the role of GAPDH phosphorylation during ground squirrel torpor.

GAPDH tyrosine phosphorylation has been previously reported in rabbit muscle but no enzymatic effect was associated with this phosphorylation event (Sergienko et al., 1992). Bioinformatic analysis of potential phosphorylation motifs within the GAPDH primary amino acid sequence did, however, indicate a phosphorylation motif that matched the insulin receptor kinase conserved motifs, possibly substantiating some link between GAPDH regulation by tyrosine phosphorylation and fuel metabolism (Appendix S2; Obenauer, Cantley & Yaffe, 2003). Additionally, GAPDH serine/threonine phosphorylation has also been well established as an important regulatory mechanism (Kawamoto & Caswell, 1986; Reiss et al., 1996; Choudhary, De & Banerjee, 2000; Tisdale, 2002; Singh et al., 2004; Tisdale & Artalejo , 2007; Boulatnikov et al., 2008; Baba et al., 2010; Huang et al., 2011). Bioinformatic assessment of the potential serine/threonine phosphorylation sites suggest PKC and CaMK as likely modifiers of GAPDH (Appendix S2), which coincides with the results observed in this study (Fig. 5). Taken together, it appears that GAPDH phosphorylation may be a significant posttranslational modification during torpor, however the specific dynamics between tyrosine and serine/threonine phosphorylation events remains to be uncovered.

Although no phospho-peptides were detected during mass spectrometry, several peptides were found to be methylated (Table 4). Both EC and LT GAPDH contained dimethylated K213, which to our knowledge is a completely novel posttranslational modification with an unknown function within the cell. This site was also found to be monomethylated in the EC condition but not in the LT condition. Interestingly, this lysine residue within rabbit muscle GAPDH was found to be reactive towards pyridoxal 5’-phosphate, which was competitively inhibited by anions such as phosphate or arsenate (Forcina et al., 1971). This suggests that this lysine residue may be important for the catalytic activity of GAPDH, and that methylation may inhibit GAPDH function by preventing ideal interaction with inorganic phosphate (a necessary process in the GAPDH catalytic mechanism). Global lysine methylation levels were assessed by Western blot and it was determined that there was no significant difference between lysine methylation states between EC and LT GAPDH (Fig. 4). Thus, it appears that lysine methylation is not responsible for the alteration in GAPDH activity during ground squirrel torpor. Alternatively, lysine methylation has been known to affect protein–protein interactions, and subcellular localization for other proteins in the cell (reviewed in Huang & Berger, 2008), and may well mediate these properties of GAPDH during euthermia and torpor within the ground squirrel.

Although lysine methylation has been fairly well characterized for a number of cellular proteins, methylation on other residues, such as aspartate, are less well known. This study identifies a novel aspartate methylation site at D242 (Seo et al., 2007). This modification was only observed for EC GAPDH, as the peptide that would contain the modification was not detected on MS/MS analyses on LT GAPDH (Table 4). Given that global levels of GAPDH aspartate methylation were not investigated and the lack of quantitation and sequence coverage from MS/MS, little can be correlated with this novel posttranslational modification. However, the prevalence and diversity of posttranslational methylation on GAPDH does provide a stepping-stone for future biochemical characterization of this and other cellular proteins.

An emerging mechanism for the regulation of cellular processes, which appears to rival posttranslational phosphorylation, is reversible protein acetylation. Indeed, recent research into this posttranslational modification indicates that it is a critical regulator of many cellular pathways, including virtually all aspects of cellular metabolism (Zhao et al., 2010; Wang et al., 2010). Acetylation has even been shown to be a prevalent and important modifier of GAPDH activity. For instance, *Arabidopsis thaliana* GAPDH acetylation was responsible for decreasing enzyme activity (Finkemeier et al., 2011). Moreover, GAPDH acetylation was found in the bacteria, *Salmonella enterica*, and human tumor cells—shifting the directionality of the enzyme towards the glycolytic direction (Guan & Xiong, 2011; Guarente, 2011; Li et al., 2014). Thus, to further characterize the regulatory properties of GAPDH, global acetylation levels were assessed for this enzyme from EC and LT conditions. While GAPDH activity did change between EC and LT conditions in this study, global acetylation levels were not altered between the two conditions. Thus, it appears that lysine acetylation does not have a significant effect on GAPDH activity during torpor.

## Conclusion

This study has characterized the kinetic, physical, and regulatory properties of thirteen-lined ground squirrel skeletal muscle GAPDH from both euthermic and torpid conditions. Kinetic analyses of this enzyme have indicated a stable suppression of GAPDH activity during torpor, which coincides with the decrease in glycolytic and gluconeogenic output observed in this tissue during dormancy. The mechanism by which GAPDH enzyme activity is stably suppressed was not conclusively elucidated in this study but may be the result of reversible phosphorylation or differential protein methylation. Further investigation into the role of GAPDH posttranslational modifications is crucial to understanding the regulation of this ubiquitous enzyme in the hibernating thirteen-lined ground squirrel.

## Supplemental Information

10.7717/peerj.634/supp-1 Appendix S1 The deduced amino acid sequence for 13-lined ground squirrel EC (A) and LT (LT) GAPDH, with sequences that were identified for ACR GAPDH by mass spectrometry highlighted in yellow

10.7717/peerj.634/supp-2 Appendix S2 Scansite 2.0 phosphorylation site prediction for thirteen-lined ground squirrel GAPDH

10.7717/peerj.634/supp-3 Data S1 All raw data for skeletal muscle GAPDH study This excel file represents all of the raw data for the present study on the properties of skeletal muscle GAPDH during mammalian hibernation.
