# Supplementary material for: Purification and properties of glyceraldehyde-3-phosphate dehydrogenase from the skeletal muscle of the hibernating ground squirrel, Ictidomys tridecemlineatus
Source: PeerJ. 2014 Oct 28;2:e634. doi: 10.7717/peerj.634 (PMC4217184; doi:10.7717/peerj.634)
Supplement: Appendix S1 [file peerj-02-634-s001.docx]

**A**

1 M V K V G V N G F G R I G R L V T R A A

21 F K S G K V D I V A I N D P F I D L N Y

41 M V Y M F Q Y D S T H G K F N G T V K A

61 E N G K L V I N G K S I S I F Q E R D P

81 A N I K W G D A G A E Y V V E S T G V F

101 T T M E K A G A H L K G G A K R V I I S

121 A S S A D A P M F V M T V N H E M Y D N

141 S P K I I S N A S C T T N C L I P L A K

161 V I H D N S S I V E G L K T T V L V I T

181 T N Q K T V D G P S G K L W H D G D G A

201 A Q N I I P A S T G A A K A V G K V I P

221 R L N G K L T G M A F R V P T P N V S V

241 V D L T C R L E K A A K Y D D I K K V V

261 K Q A S E G P L K G I L G Y T E D Q V V

281 S C D F N S D I H S S T F D A G A G I A

301 L N D H F V K L I S W Y D N E F G Y S N

321 R V V D L M V H M A S K E *

**B**

1 M V K V G V N G F G R I G R L V T R A A

21 F K S G K V D I V A I N D P F I D L N Y

41 M V Y M F Q Y D S T H G K F N G T V K A

61 E N G K L V I N G K S I S I F Q E R D P

81 A N I K W G D A G A E Y V V E S T G V F

101 T T M E K A G A H L K G G A K R V I I S

121 A S S A D A P M F V M T V N H E M Y D N

141 S P K I I S N A S C T T N C L I P L A K

161 V I H D N S S I V E G L K T T V L V I T

181 T N Q K T V D G P S G K L W H D G D G A

201 A Q N I I P A S T G A A K A V G K V I P

221 R L N G K L T G M A F R V P T P N V S V

241 V D L T C R L E K A A K Y D D I K K V V

261 K Q A S E G P L K G I L G Y T E D Q V V

281 S C D F N S D I H S S T F D A G A G I A

301 L N D H F V K L I S W Y D N E F G Y S N

321 R V V D L M V H M A S K E *
