# Supplementary figures and images for: Purification and properties of glyceraldehyde-3-phosphate dehydrogenase from the skeletal muscle of the hibernating ground squirrel, Ictidomys tridecemlineatus
Source: PeerJ. 2014 Oct 28;2:e634. doi: 10.7717/peerj.634 (PMC4217184; doi:10.7717/peerj.634)

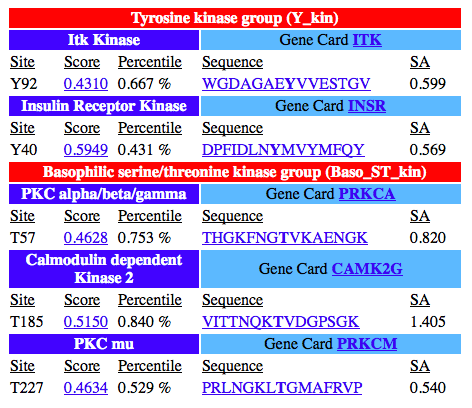

Supplement: Appendix S2 [file peerj-02-634-s002.docx]
